# Could the medical humanities be a remedy for the rising disengagement between physician and patient?

**DOI:** 10.15694/mep.2018.0000179.1

**Published:** 2018-08-21

**Authors:** Cristóbal Pera, Manuel Pera

**Affiliations:** 1 University of Barcelona; 2 Autonomous University of Barcelona

**Keywords:** Patient-Doctor Relationship, Technology, Dehumanization, Human Dignity, Medical Humanities.

## Abstract

This article was migrated. The article was marked as recommended.

The idea of incorporating an interdisciplinary field about the Human Condition of the patient and its intrinsic dignity, under the title of Medical Humanities or simply Humanities into the core curriculum of medical education seems reasonable. We hope it may become a reality as remedy for the difficulties of the doctor-patient relationship in modern medicine, which is extremely technological.

## Perspective

### The difficulties of the doctor-patient relationship in modern medicine

“An educational ideal, in which humanism and the realism of the natural sciences are joined to one another, for their mutual enlightenment, has not been realized so far”

K. Jaspers (1959) *The Idea of the University*, Boston: Beacon Press.

Nowadays, for the majority of citizens there is growing concern, if not frank discontent, with the slow ritual that precedes a first requested appointment with the doctor. Mainly, this is due to the lack of harmony with how this very special relationship between the patient seeking help and the doctor, who is presumably ready to lend it, usually starts.

As a consequence of this worrying mismatch between patient and doctor, critics are becoming increasingly concerned about the growing bias, technological, impersonal and bureaucracy of clinical practice.

In this fuzzy context, within the medical act, simple only in appearance, where the doctor observes and listens to the patient, while he relates what ails and what worries him (disease versus illness), the happy expression “medical gaze” ( *regard medical*, as it was used originally by Michel Foucault), which is inquisitive and, at the same time, compassionate, is being neglected by most doctors ( Foucault, 1994).

To qualify this disagreementbetween the protagonists, the most commonly used term now is dehumanization as it deprives the patient of his human attributes and, principally of their Human Dignity, an intrinsic merit that “conditions” the progression from Human Nature to Human Condition ( Arendt, 1998). Dignity as an intrinsic value of the Human Condition, a conceptual figure that is accessed through three facets - ethical, legal and political - that postulates not only the right to live, but also to some conditions where it is worth living.

A dignity that is perhaps more easily and better understood from the negative side, for the horror caused by the sad spectacle of its transgression (alienation ofthe strange, persecution, humiliation, slavery, starvation, extermination, holocaust), than from the positive side, as academic definition of this human right ( Andorno, 2011).

If, by contrast, the accent is on its consideration as a simple object devoid of his identity as a person, in your individuality, the disagreement between the two – patient and doctor - is qualified as depersonalization.

A clear disregard for the patient thus treated because the doctor-patient relationship is a human task, with two bodies involved, both with their Human Condition.

• Human Condition – after Hanna Arendt- “is not the same as human nature, and the total sum of human activities and capabilities which correspond to the human condition does not constitute anything like Human Nature.”

The Human Condition comes from Human Nature, with its resonance in the first words naming the things of the world, with which it is woven into the language, and thought is elaborated and deployed, inquisitive or creative, into one’s own body, asking themselves, or thinking out towards “the state of things” or “state of affairs” in the world ( Husserl, 2001).

Of the words of the human body, when they are used inside the body, emerges the consciousness of being in the world, as a person, which when expelled facilitate real interaction between people, “without the intermediary of things or matter” ( Arendt, 1998).

The dehumanization of the patient-doctor relationship has been provoked, largely, by the extraordinary scientific and technological advances of Modern Biomedicine, with its impressive panoply of available diagnostic procedures that seem to completely surpass, in terms of their results, those of the clinical interview, a human encounter - based on the mutual trust generated by the hand and the word - to the point of considering it unfairly obsolete.

In addition, the overload of the health public systems helps to minimize the crucial role of the conversation with the patient, while the integrative medical gaze has been replaced by cold communication through technological data and images.

The great scientific and technological achievements of modern medicine and the commoditization of the health services, with their very diffuse boundaries between public and private, overload doctors’ agendas that work in both systems. To such an extent that they do no longer talk with their patients, and when they are forced to answer your doubts and fears, they do so in a dry and impatient way, because they are always in a continuous state of haste. Under those conditions it is difficult that the relationship between patient and doctor can develop as a moral activity based on a pact of trust ( Arendt, 1998).

### Introduction of humanism into the core of medical education

“For this is *humanism*: meditating and caring, that human beings be human and not inhumane, “inhuman,” that is, outside their essence. But in what does the humanity of the human being consist? It lies in his essence”.

M. Heidegger. (1949) *Letter on “Humanism”,* Cambridge University Press****

With the introduction of Humanism into Medical Education, both pre- and postgraduate, under the title of Medical Humanities, with the purpose of tackling the progressive and harmful disagreement between patient and physician, causing the dehumanization of the first, there arise a number of questions ( Pera, 2001):

What humanism is being spoken of?

• Humanism as paideia?
• Humanism as history of the different contexts of the relation between patient and physician?
• Humanism as ideology centred, for better or worse, in the human being?
• Humanism as ethics concerned on respect for the human dignity?
• Humanism as a philosophy that places the accent on the existence of the human being and in the primacy of his subjectivity (Psychology) that settles in the body, from which they sprout words, thoughts, feelings and the awareness of being in the world?
• Humanism as art who interprets, from different visions, the real and the imaginary?
• Humanism as adornment, closest to mere erudition?
• Humanism with special concern and respect for the human being, above all of his dignity as a person?

Our answer is that we must take into account all these types – paideia, history, ideology, ethics, philosophy, psychology, arts, and human dignity - except for the humanism as simple adornment.

However, a clear definition of the real objective of the incorporation of Medical Humanities into the core curriculum of the medical education, as well as a delimitation of its content is not easily found in most published papers ( Macnaughton, 2000). The majority are too extensive, with imprecise field limits, while some of the more elaborate are far too short and cryptic.

Two examples of both definition styles **:**

“The Medical Humanities provide an interdisciplinary and interprofessional approach to investigating and understanding the profound effects of illness and disease on patients, health professionals, and the social worlds in which they live and work. In contrast to the medical sciences, the medical humanities - which include narrative medicine, history of medicine, culture studies, science and technology studies, medical anthropology, ethics, economics, philosophy and the arts (literature, film, visual art) - focus more on meaning making than measurement.”( Baylor University, 2018)

“We define Medical Humanities as an inter- and multidisciplinary field that explores contexts, experiences and critical and conceptual issues in medicine and health care, while supporting professional identity formation.” (Cole, Carlin and Carson, 2015)

The majority of those speaking and writing under this imprecise title of Medical Humanities recommend its incorporation into the core curriculum of medical education. However, in our opinion, the main problem is that they do not define clearly the fundamental objective that is, from our point of view, to remedy the serious mismatch between patient and doctor.

To this serious obstacle we could add that the so-called Medical Humanities have not been “thought from the body” ( Pera, 2006), but from a Cartesian sense, without bearing in mind that everything that encloses the patient human condition – words, thoughts, feelings, emotions and consciousness as a person in the world - spring from the body, a very complex biological space, vested with dignity, as an intrinsic value as a moral person ( Kant, 2012).

To remedy the raising disengagement between patient and physician, with its consequent loss of confidence, the incorporation of Medical Humanities, as a set of disciplines that revolve around the human being, like literature, philosophy, ethics, psychology, history and art, into the core curriculum of medical education, could be the answer. This does however depend on it being seen and, thus organized, as a field of open discussion about the human side of the medical act and not from the closed and inoperative concept of a subject within the domains of a department.

We believe in a field of knowledge, complementary, but enriching, for medical education that, through open dialogue, may stimulate within the students the development of free and critical thinking. A thinking that will be capable of asking questions about the problems that affects the human being in today’s world, when the intrinsic dignity of his body is ignored, as unfortunately too often happens in the patient-doctor relationship.

In the end, the fundamental goal of the introduction of the so-called Medical Humanities into the core curriculum of Medical Education is to humanize the vital encounter between patient and physician.

## Conclusion

***Between the idea***

***And the reality***

***Between the motion***

***And the act***

***Falls the Shadow***

TS Eliot, The Hollow Men,

Selected Poems, (1976) FABER AND FABER,London

The idea of incorporating an interdisciplinary field about the Human Condition of the patient and its intrinsic dignity, under the title of Medical Humanities or simply Humanities into the core curriculum of medical education seems reasonable. We hope it may become a reality as remedy for the difficulties of the doctor-patient relationship in modern medicine, which is extremely technological.

Michael Lam and colleagues in a paper entitled “ *A review of medical humanities curriculum in medical schools”* reach the following conclusion ( Lam M *et al.*, 2015):

“Given the abundant literature discussing the merits of humanities, it is important that medical humanities be incorporated into medical school curricula locally as well as globally.”

Although he adds:

“To fully assess the impact of student selection and curriculum development, a long-term longitudinal study will be required in order to reveal whether today’s new practices have in fact assisted in the making of a good doctor”.******

## Take Home Messages

• Medical Humanities as a field of knowledge, complementary, but enriching, for medical education that, through open dialogue, may stimulate within the students the development of free and critical thinking.
• The fundamental goal of the introduction of the so-called Medical Humanities into the core curriculum of Medical Education is to humanize the vital encounter between patient and physician.

## Notes On Contributors

Cristóbal Pera [Hon FRCS (Engl)] is Professor Emeritus of Surgery of the University of Barcelona and former Chairman of the Department of Surgery at the Hospital Clinic (Barcelona, Spain). He served as Dean of the School of Medicine and he organized a prestigious program of medical humanities during the early nineties. He authored a well-known Textbook of Surgery (“Surgery: Fundaments. Indications and Technical Options”) and has published several books and essays about the body, highlighting, amongst others, “El cuerpo herido: un diccionario filosófico de la cirugía” [The wounded body: A philosophical dictionary of surgery”] (Acantilado, 2003).

Manuel Pera is the Head of the Section of Gastrointestinal Surgery at Hospital del Mar, Barcelona, Spain, and Professor of Surgery at the Autonomous University of Barcelona, Barcelona, Spain. In 2011 he founded together with a group of dynamic medical students The Gimbernat Surgical Association ( www.aqgimbernat.com) to promote surgical specialities among students, incorporating since the beginning programmed dialogues about the role of humanism in Medical Education, particularly in the patient-physician relationship.
