# Supplementary material for: Could the medical humanities be a remedy for the rising disengagement between physician and patient?
Source: MedEdPublish (2016). 2018 Aug 21;7:179. [Version 1] doi: 10.15694/mep.2018.0000179.1 (PMC10701851; doi:10.15694/mep.2018.0000179.1)
Supplement: Peer review history

# Reviewer report: Reviewer response for version 1

**DOI:** 10.21956/mep.19630.r29221

**Published:** 2018-09-04

**Authors:** P Ravi Shankar

**Affiliations:** 1 American International Medical University

This review has been migrated. The reviewer awarded 3 stars out of 5 This is an interesting article but I found it difficult to understand in many places. I am of the opinion that medical educators and practicing clinicians should play an important role in the medical humanities (MH). It is praiseworthy that the authors who are surgeons have a keen interest in MH. One of the challenges with involving doctors more in MH education would be to help doctors make sense of MH and make them more comfortable with the subject. The authors could describe in greater detail how MH could address the rising disengagement between the patient and the doctor. This is the title of their manuscript and I am of the opinion that they may have to address this in greater detail.

Reviewer Expertise:

NA

No decision status is available

# Reviewer report: Reviewer response for version 1

**DOI:** 10.21956/mep.19630.r29220

**Published:** 2018-08-23

**Authors:** Jonathan McFarland

**Affiliations:** 1 Sechenov University, Moscow

This review has been migrated. The reviewer awarded 5 stars out of 5 There are good articles and there are unique articles. This, as well as being a good and very thought-provoking article, is also unique in that the two distinguished authors are both surgeons and father and son. It was a true pleasure to read this article taking this fact into account. The theme of the article is topical, and that is, can the MH remedy the growing disengagement between Dr and patient. For me, it had added weight due to the enormous combined experience both authors have as doctors/surgeons. It is very important that we do get the words/advice of those at the coal face, as it were. The authors highlight some concepts, which perhaps in our fast-moving world tend to be ignored both within society and within medicine: dignity, for should this not be at the forefront of all human relationships?, the difference between illness and disease ( in some languages - Spanish and Russian for example there is only one word for these two), and the "happy expression "medical gaze", which is both compassionate and inquisitive, as Foucault mentions. These concepts need to be at the core of medical training and practice. I also found the emphasis on the patient-Dr relationship to be extremely pertinent, as isn't this what medicine is all about ? Sometimes, I dare say, this can be forgotten. All in all, I found this to be a beautiful and intellectually moving paper, which should be read by all those who are devotees of bringing back humanism into medical education. Perhaps there could have been a little more on "how" but then again that is the million and one question, which we all need to bring our heads together to try and resolve. Thank you Professors Pera.

Reviewer Expertise:

NA

No decision status is available

# Reviewer report: Reviewer response for version 1

**DOI:** 10.21956/mep.19630.r29219

**Published:** 2018-08-21

**Authors:** Trevor Gibbs

**Affiliations:** 1 AMEE

This review has been migrated. The reviewer awarded 3 stars out of 5 An interesting paper that explores some of the fuzzy edges of the Humanities and tries to draw together some of the various definitions that we use. I am not sure that many would disagree with the words and feelings expressed, they have been represented before in many different guises - in this paper they are very well expressed and show the inner belief within the two authors. My concern and I suppose now my need, is to see these words transferred into action, how do we effectively place the Humanities into our curriculum, which is the best way, do we create stand-alone courses or integrate the humanities into all subjects and all years, how can we evaluate its real effect on our students, are we producing "better" doctors and if we are what do we mean? - many questions still abound.So although I would recommend this paper to be read by those interested in the humanities, I think that it could have been improved by perhaps attempting to answer some of these important questions that we now have.

Reviewer Expertise:

NA

No decision status is available
